# Factors affecting sick leave prescribing in occupational health care: a survey based on hypothetical patient cases

**DOI:** 10.1186/1472-6963-14-168

**Published:** 2014-04-14

**Authors:** Anni T Kankaanpää, Tuula M Putus, Risto J Tuominen

**Affiliations:** 1 Department of Public Health, University of Turku, and Turku University Hospital, Turku, Finland; 2 Department of Occupational Health Care, University of Turku, Turku, Finland; 3 Department of Public Health, University of Turku, and Primary Health Care Unit, Turku University Hospital, Turku, Finland

**Keywords:** Sick leave, Occupational health care, Clinical medicine, Sickness absence, Health services research

## Abstract

**Background:**

Several studies have shown considerable differences in the way that physicians prescribe sick leave. The aim of this study was to examine the sick leave prescribing practices of occupational health care physicians and factors affecting these practices.

**Methods:**

A questionnaire study with 19 hypothetical patient cases was conducted among 356 Finnish occupational health care physicians. The effects of both physician-related and local structural background variables on sick leave prescribing were studied using regression models. Economic consequences of the variation in sick leave prescribing were estimated.

**Results:**

When the cases were considered individually, the variation in prescribed sick leave days was relatively small. However, when considered together, variation in prescribing practice became apparent. On average, the overall number of days of sick leave prescribed for the entire group of 19 patient cases was 85.8, varying between 21 and 170 days. The physicians working at a public health center and those with more than 20 years experience as an occupational health physician tended to prescribe more days of sick leave than others. The quartile of physicians who prescribed the fewest days of sick leave would have resulted in mean production losses (17,100 euro, 95% CI 16,400-17,700) that were half those in the quartile with the most days of sick leave (34,800 euro, 95% CI 33,600-35,900).

**Conclusions:**

There was variation in the sick leave prescribing practices of occupational health care physicians. The most significant factor affecting this variation was the health care sector (public, private or employer clinic) employing the physicians. Variation in sick leave prescribing patterns can lead to inequality between patients.

## Background

Sickness benefits reduce the economic burden on an individual with reduced work capacity due to illness by transferring the cost of lost productivity to third parties such as employers or the state. In medical practice, sick leave has been estimated to be the single most expensive component of costs, even more expensive than drug prescriptions [1,2].

In Finland, all people are insured by the public social insurance system that is financed through taxation. The costs of the first ten days of absence from work due to illness (sick leave) are covered by employers; the employer is responsible for paying full salary to the employee on such short term sick leave. The national social insurance system covers the wage losses after the tenth day of sick leave. Although information exists on social insurance payments for sick leave on the tenth day and onwards, there are no registers or readily available databases on shorter periods of sick leave.

In the Finnish health care setting, all physicians (general physicians, occupational health care physicians, physicians in a service of an employer etc.) are equally qualified to prescribe sick leave, and it is a part of every physician’s daily clinical practice. Thus physicians have a central role as gatekeepers of the social security system in order to provide sickness benefit only to those who have a medically acceptable cause of sickness absence [3,4]. On the other hand the physician should ensure that the patient obtains his or her rightful part of the common resources, as the physician is the patient’s advocate [3,4]. In Finland occupational health care services are provided in public health centers to a limited extent determined by law. These physicians mainly perform regular check-ups and treat uncomplicated cases. Physicians in private practice and in employer clinics provide these services and more comprehensive care than their colleagues in the public sector.

Primary care physicians regard the tasks of sick-listing and work ability assessments as problematic and some of the most challenging in their practice [4-7]. When sick-listing a patient, the physician is influenced by a number of factors other than purely medical considerations [2,3,8]. Several studies indicate that if a patient initiated the discussion around sickness certification, the physician nearly always sick-listed the patient [4,8-10]. The most important factor for sick-listing was the patient’s way of presenting his or her problem [11]. In most cases, the physician has to rely on the patient’s description of work tasks. The physician’s knowledge of a patient’s work demands is thereby second-hand knowledge [12]. Occupational health care physicians might handle the situation better than primary care physicians because of their knowledge of and contact with work places, which might give them a better basis for the decision when evaluating the patient’s ability to work [3].

The few studies that have analyzed sick leave prescribing practices have shown variation between physicians [1,3,4,11,13,14]. A Swedish population-based study showed that GPs sick-listed patients for shorter periods than other physicians [3]. Occupational health care physicians used partial sick-listing more than other physicians [3]. Older age and extensive working experience of a physician seem to correlate with prescribing more days of sick leave [2,15,16]. Primary care physicians practicing in large municipalities prescribed shorter sick leaves than others in a Finnish study [13], but a Swedish study reported contradictory findings [14]. The effect of the gender of a physician on sick leave prescribing practices has been ambiguous: in most studies male and female physicians prescribed equal amounts of sick leave [1,2,13,15], but variation has also been found [9,11]. The effect of physician-related factors and local structural factors on prescribing practices also appears to be ambiguous. Some studies suggest that these factors may influence the length of sick leave [2-4,8,9,11,13-16], but other studies have found no associations [1,2,4,13,15].

The purpose of this study was to examine the prescribing of sick leave by occupational health care physicians, the extent of any variation in practices, and whether physician-related factors and local structural factors contribute to any variation.

## Methods

The survey was based on a sample of 683 occupational health care physicians (every second occupational health care physician working in Finland) drawn from the membership register of the Finnish Medical Association. A pre-tested questionnaire was sent to the sample of physicians in February 2011. Non-respondents were sent a second questionnaire two months after the dead-line for returning the first questionnaire.

At the beginning of the questionnaire, the purpose of the study was described and the recipients were told that participation was voluntary. The first set of questions covered socio-demographic background information: age, sex and the postal code of the catchment area of the municipality where the physician mainly worked. This was followed by questions dealing with factors related to work and training: years worked in occupational health care, degrees in a medical specialty, specialty areas, main working sector, numbers of working hours per week as a physician in general and in the occupational health care, and the size of any hospital in the municipality where the physician mainly worked.

Next followed 19 hypothetical case simulations representing typical patients seen by occupational health care physicians. The cases were created in co-operation with experienced specialists in occupational health care and family practice. All cases included information about the patient’s age, sex, occupation, brief description of symptoms and the ICD-10 diagnosis, for example: *Patient: 23-year-old female, barmaid. Symptoms: Typical common cold, no fever. Diagnosis: J06.9 Acute upper respiratory infection.* The cases were described in brief so that they would be representative of the patients seen by occupational health care physicians [Additional file 1]. The physicians were asked to consider each case as a typical non-complicated patient attending an initial visit on a Monday morning, and to evaluate how many days of sick-leave he/she would prescribe to each patient according to his/her usual practice. Eighteen different diagnoses were chosen for the cases; two patients had the diagnosis of depression: a 26-year-old woman and a 45-year-old man. In the very few cases where some respondents answered with a range of days, the mean value was used in analyses.

For the analyses, the variables were categorised [Table 1]. The physician’s clinical working experience in occupational health care in years was used both as a continuous variable and dichotomized. The main working sector of the physician was classified and a dichotomy was formed (working in public health center, or other). The population of the catchment area of the main working municipality was collected from Statistics Finland based on the postal code. This was used as categorized variable, continuous variable and dichotomized (small area of 100,000 or less inhabitants, or large area of more than 100,000). The number of days of sick leave for each simulated case was studied separately, and an overall variable was formed by summing up the days of sick leave each physician prescribed for the 19 cases.

**Table 1:** Background characteristics of the 356 participating occupational health care physicians

**Variable**	**%**	**n**
Having a specialty degree		
Yes	69.7	246
No	30.3	107
In occupational health care	41.6	148
Clinical working experience in occupational health care		
20 years or less	76.7	267
More than 20 years	23.3	81
Main working sector		
Public health center	12.0	42
Private practice	53.8	189
Employer clinic	12.8	45
Other	21.4	75
Population of the catchment area of the main working municipality		
20,000 inhabitants or less	9.4	31
21,000 to 50,000 inhabitants	15.4	51
51,000 to 100,000 inhabitants	21.1	70
More than 100,000 inhabitants	54.1	179
Hospital in the main working municipality		
University hospital	52.4	184
Central hospital	26.5	93
District hospital	11.7	41
Health center hospital	8.3	29
None	1.1	4

To estimate the possible economic consequence of sick leave prescribing practices from the societal perspective, the age, sex and occupation-specific mean salaries from official statistics were used for each patient case. Because all cases required relatively short sick leaves, the effects of possible social security reimbursements were not applicable. The production losses of the sick leave prescribing practices were estimated by applying the computation formula of the Confederation of Finnish Industries: total societal productivity loss of a day of sick leave equals three times the daily gross salary.

The statistical evaluation of the data was based on chi-square test for proportions and Student’s *t*-test for means. Pearson’s coefficients of correlation were used to examine the degree of relationship between two continuous variables. Univariate linear regression models and a multiple linear regression model were fitted to study the effects of the selected background variables on the overall number of sick leave days the physician prescribed for the 19 patient cases.

Because the questionnaire was directed to practicing physicians and hypothetical patient cases were described, the ethical committee of University of Turku and the Hospital District of Southwest Finland decided that their approval was not required.

## Results

The final number of returned questionnaire was 356 representing 52.1% of the sample. A majority (63.1%) of the physicians who participated in the study was female. The average age was 49.3 years (SD 8.4), 69.7% had a specialty degree, of which 63.5% were in occupational health care, and 67.8% worked only in occupational health care. The distributions of other background variables are presented in Table 1.

When considering the cases individually, the variation in prescribed sick leave days was relatively small. However, when all the cases were considered together, considerable variation in prescribing practices became apparent. On average, the overall number of days of sick leave prescribed for the entire group of 19 patient cases was 85.8, varying between 21 and 170 days. The total days of sick leave for the 19 patient cases prescribed by physicians working at a public health center was greater than by those working in private practice or at an employer clinic [Table 2]. Four cases (both depression cases, pneumonia and wound of the hand) received large amounts of sick leave compared to other cases. However, when these cases were excluded from the analyses, the differences in the total amount of sick leave days between physicians working in different sectors did not reach statistical significance.

**Table 2:** Mean number and range of days of sick leave for 19 hypothetical patient cases classified by the physicians’ working sector

**Diagnosis**	**Public health center, n = 42**		**Private practice, n = 189**		**Employer clinic, n = 45**		**All physicians, n = 351**	
	**Mean**	**Range**	**Mean**	**Range**	**Mean**	**Range**	**Mean**	**Range**
Lumbago	4.9	2-10	4.3	2-10	4.5	3-14	4.4	2-14
Tension headache	2.2	0-6	2.3	0-5	2.2	0-7	2.2	0-7
Tennis elbow	6.7	3-14	6.3	0-14	7.0	0-14	6.6	0-14
Common cold	2.3	0-3	2.3	0-4	2.3	0-3	2.3	0-5
Pneumonia	9.1	5-21	8.4	2-15	9.1	1-20	8.7	1-21
Gastroenteritis	2.6	1-5	2.4	1-7	2.4	1-5	2.5	1-7
Shingles	4.9	0-10	4.8	0-14	4.9	0-10	4.8	0-14
Vertigo	3.1	0-10	3.0	0-11	2.9	0-7	3.0	0-11
Wound of a hand	8.1	5-14	7.4	0-14	6.9	1-15	7.3	0-15
Sprained ankle	6.6	3-14	6.4	1-21	6.2	2-12	6.5	1-21
Burn	4.3	0-10	4.5	0-14	4.2	0-14	4.5	0-14
Atopic eczema	1.6	0-14	1.1	0-10	0.6	0-7	1.1	0-14
Contractions	4.5	0-14	3.8	0-14	2.9_a_	0-7	3.7	0-14
COPD	4.3	0-9	3.7	0-10	3.3_a_	0-7	3.8	0-10
Depression, female	11.1	0-30	9.3	0-30	10.1	0-30	9.8	0-30
Depression, male	12.0	0-30	10.1	0-30	10.8	0-30	10.6	0-30
Stress	2.2	0-14	1.9	0-18	1.8	0-7	1.9	0-18
Hangover	0.6	0-4	0.5	0-3	0.4	0-3	0.5	0-4
Cystitis	0.9	0-5	0.8	0-3	0.6	0-2	0.8	0-5
All 19 cases together	94.1	46-161	84.1^a^	21-170	83.7	38-125	85.7	21-170

Statistical comparisons of private practice and employer clinic compared to public health center physicians.

^a^p < 0.05, One-way ANOVA, pairwise comparison with Dunnett’s method using public health center as a control group.

The amount of years worked as an occupational health physician correlated positively with the amount of sick leave days prescribed for the 19 cases together (p < 0.05). For individual cases, the correlation was positive in 15 cases with the correlation reaching statistical significance in three cases. The physicians with 20 years or less working experience in occupational health care prescribed 83.9 (SD 22.2) days of sick leave to all the patients together, while the physicians with more than 20 years of experience prescribed 91.5 (SD 25.3) days (p < 0.05). Having a specialty degree did not have an effect on the amount of sick leave prescribed, and the prescribing practices of the specialists in occupational health care did not differ from those of other specialists.

Physicians working in smaller (<100,000 inhabitants) municipalities prescribed more days of sick leave (88.2) to all the patient cases together than those working in larger municipalities (83.0), (p = 0.05). The difference was also significant for four individual cases.

On average, the economic consequence to society of the sick leave prescribed to all 19 cases would be 25,600 euro (95% CI 24,800-26,400). The sick leave prescribed by a physician from the quartile of physicians who prescribed the fewest days of sick leave would have resulted in average production losses of 17,100 euro (95% CI 16,400-17,700), while that of the quartile with the most days of sick leave would be 34,800 euro (95% CI 33,600-35,900).

There was a tendency for male physicians to prescribe slightly more sick leave days (88.4) to all the 19 patient cases together than female physicians (84.3), though this difference did not reach statistical significance (p = 0.127). For male patients, male physicians did not prescribe shorter or longer sick leave than female physicians, but for female patients, male physicians tended to prescribe slightly longer sick leave (49.5 days) than female physicians (46.8 days), though this was not a statistically significant difference (p = 0.087).

In the univariate linear regression models, working experience of more than 20 years, working in a public health center and working in a smaller municipality showed significant association with the prescribing of higher number of sick leave days [Table 3]. When multiple linear regression model with these three variables was conducted, only the effect of working experience remained significant (R^2^ = 0.015). Collinearity testing showed that the role of age was non-significant and longer working experience had an independent effect.

**Table 3:** Univariate linear regression models of selected background variables on total days of sick leave for all 19 cases

	**Univariate models**		
**Variable**	**β**	**SE**	**p =**
Age in years	0.190	0.152	0.213
Dichotomies			
Male sex	4.110	2.683	0.127
Experience more than			
20 years	7.580	3.073	0.014
Municipal population			
> 100,000	−5.196	2.642	0.050
Specialty degree	−1.538	2.778	0.580
Working in public health center	9.509	3.960	0.017
Central hospital in working municipality	1.067	3.161	0.736

## Discussion

The prescribing of sick leave varied between physicians. The variation was relatively small for most cases considered individually, probably because the sick leave periods involved were short. However when the total amount of sick leave prescribed for all cases together was examined, the differences between physicians’ prescribing practices emerged. The patient cases described for the physicians were developed with clinical specialists who had long experience in general and occupational medicine, with the aim of representing typical patients seen in everyday practice. The findings indicate that systematic prescription of slightly longer sick leave to individual patients results in a significant increase in the accumulated days of sick leave.

The most significant physician-related factor affecting the variation in prescribing of sick leave was the type of main working sector. Physicians working in employer clinics and in the private sector prescribed shorter sick leaves to the hypothetical patient cases than those working in public health centers. In public health centers access to care and possible reappointment may be more difficult to arrange than in the private sector and employer clinics because of long queues [17]. This might affect the treatment practices of physicians in the public sector, as they may prescribe longer sick leaves for good measure.

It has been argued that “Sickness certification may be regarded as a drug with effects and side-effects, it should be used in proper doses, and thus should be handled in the same way as a medical prescription” [15]. However, there is no strong medical evidence of the benefit of rest or staying at home from work [4,11]. In Finland, there are no guidelines for physicians on appropriate prescribing of sick leave, and estimating the need for or adequate length of sick leave is not included in the curriculum of medical students. All prescribing of sick leave is solely dependent on the experience, impressions and customs of each individual physician. In particular, physicians at the beginning of their clinical career may face difficulties deciding how many days of sick leave could be relevant in each patient case. Education on sick leave prescribing could be emphasized both in undergraduate and postgraduate training. In Finland there is no return to work certificate but the patient returns to work when sick leave days on the initial sickness certificate have passed. If the patient remains incapable of working, a new work ability assessment can be conducted and the sick leave period with sick leave compensation can be continued.

In our previous study we used the same 19 patient cases to examine the sick leave prescribing practices of primary care physicians [13]. The average number of sick leave days they prescribed (97.4 days) was more than that prescribed by the physicians in this study. One reason for the difference might be that occupational health physicians, who work in close co-operation with the work places, can offer options to modify and lighten the patient’s work tasks or to arrange work rehabilitation, so that the patient can return to work earlier. The cost containment of occupational health physicians may be due to their co-operation with employers as occupational health care physicians may feel that employers expect them to try to obviate employees’ absence from work when possible.

Two earlier Swedish studies have presented opposing results to ours, reporting that general practitioners prescribe shorter sick leaves than occupational health physicians [3,14], and that physicians practicing in large municipalities prescribed longer sick leaves than others [14]. A different study setting may be the explanation. In the Swedish studies long-term sick leave was also included and register data was used, contrary to our setting with short-term sick leave and hypothetical patient cases. Comparing results from different countries is problematic due to differences in organization of systems for benefit claims.

There was a normal distribution in the sick leave days for all cases. Significant correlation was observed between some of the background variables studied. Univariate analyses showed significant associations with the numbers of sick leave days, including situations where the variable under study was actually reflecting the effect of another, correlating variable. The β coefficient of age indicates that on average one additional year of age means 0.19 days more sick leaves prescribed. The coefficients of dichotomous variables indicate how the condition mentioned increase (positive coefficients) or decrease (negative coefficient) the overall number of prescribed sick leave days.

Older age and extensive working experience of a physician seem to correlate with greater amount of sick leave days [2,15,16]. Our results confirm the finding concerning the working experience but not the age. The effect of the gender of a physician on sick leave prescribing practices is unclear: in most studies male and female physicians prescribed equal amounts of sick leave [1,2,13,15], but variation has also been found [8,9,11]. The variation found in our research was minor and non-significant.

According to our findings, the estimated costs to society of prescribed sick leave can be considerable. The difference between the least and most costly prescribing practice by occupational health care physicians was almost ten-fold. This estimate corroborates the earlier finding that sick leave prescribing causes significant economic consequences to the society [1,2].

Because Finnish National Social Insurance registers only sick leave after the tenth day, a case simulation study design was chosen to enable investigation of shorter sick leave prescribing practices, and to standardize the descriptions of the symptoms and patient characteristics. The respondents were asked to assume each patient case was seen at an initial visit on a Monday morning, so that the basis for the assessments would be as similar as possible. These actions were taken to increase the reliability in depicting the variations in sick leave prescribing practices of physicians. All cases were represented as simple and not complicated, because the aim was to study the most common diseases which cause the largest amount of short sick leave periods. In real patients the diseases and the situations are often more complicated, and the variation in physicians’ practices could be greater.

Studies have confirmed that hypothetical patient cases are a valid and comprehensive tool for measuring the quality of clinical practice [18-20]. It has been proposed that this method makes the clinicians do their best, when the results represent the ideal situation without distractions [21,22]. However, using hypothetical cases has also been criticized because there is no consensus on how well the results of the case studies represent actual behaviour [22]. Due to the short case descriptions the physicians had to make their decisions with relatively little information which could have caused increased variation between physicians and some bias in results.

Essentially all Finnish physicians involved in clinical work are members of the Finnish Medical Association, the source of the sample. The sample covered the whole country and was considered large enough to run the chosen analyses. Age, sex and occupational characteristics of the participants reflected those of all practicing occupational health care physicians in Finland. Thus, these study findings can be expected to represent relatively well the views, opinions and actions of all Finnish occupational health care physicians. The response rate was over 50%, which could be considered as satisfactory in postal surveys. However, we did not have an opportunity to study in more detail the characteristics of non-respondents. Nevertheless, there is a possibility of differences due to geographic or working demands. Patients visiting different occupational health physicians might work in blue or white collar settings, which might have influence on findings.

The economic consequences for society due to sick leave prescribing practices were estimated by applying the computation formula of the Confederation of Finnish Industries [23]. In addition to the daily gross salary during the sick leave period, the formula also takes into account the economic consequences from other causes, such as production losses, time needed for finding substitutes, the expense of hiring substitutes, the lower productivity of substitutes compared to regular workers and the expenses from occupational health care. The formula is robust and is commonly used in Finland.

## Conclusions

The variation in the sick leave prescribing practices of occupational health care physicians is minor when observing individual patient cases, but systematic prescription of more sick leave days by some physicians can have significant influence. Decrease in variation might improve equality between patients. The most significant factor affecting variation in prescribing practices was the main working sector (public, private or employer clinic) of the occupational health care physicians.

## Competing interests

The authors of this manuscript do not have any financial or non-financial competing interests.

## Authors’ contributions

ATK participated in the design of the study, performed the statistical analysis and drafted the manuscript. TMP helped to draft the manuscript. RJT participated in the design of the study, helped with the statistical analysis and helped to draft the manuscript. All authors read and approved the final submitted manuscript.

## Pre-publication history

The pre-publication history for this paper can be accessed here:

http://www.biomedcentral.com/1472-6963/14/168/prepub

## Supplementary Material

Additional file 1 Cases. Click here for file
