# Supplementary material for: Factors affecting sick leave prescribing in occupational health care: a survey based on hypothetical patient cases
Source: BMC Health Serv Res. 2014 Apr 14;14:168. doi: 10.1186/1472-6963-14-168 (PMC4021310; doi:10.1186/1472-6963-14-168)
Supplement: Additional file 1 — Cases. [file 1472-6963-14-168-S1.doc]

Additional file 1. Cases.

1. 50-year-old man, carpenter. Symptoms: sudden severe backache, no abnormal neurological findings. M54.5 Lumbago.
2. 36-year-old man, bus driver. Symptoms: headache. G44.2 Tension neck.
3. 45-year-old woman, seamstress. Symptoms: pain in an elbow. M 77.1 Lateral epicodylitis.
4. 22-year-old woman, bar tender. Symptoms: typical flu, no fever. J06.9 Common cold.
5. 25-year-old man, shop assistant in an home appliance shop. Symptoms: Fever and cough, typical pneumonia-signs in thorax x-ray. You decide to treat the patient with antibiotics at home. J18.9 Pneumonia.
6. 31-year-old woman, councel. Symptoms: Vomiting and diarrhea. Patient’s children have just been vomiting too. No travelling in foreign countries in anamnesis. A08.4 Gastroenteritis caused by a virus.
7. 40-year-old woman, cleaner. Symptoms: Achy blistering rash at waist. B02.9 Shingles.
8. 39-year-old man, teacher. Symptoms: Formerly diagnosed benign vertigo with neurological examination. Now similar vertigo as before. H81.1 Vertigo.
9. 33-year-old man, car mechanic. Symptoms: Got a 4 cm long wound in the palm while working, no related muscle or tendon lesion. You close the wound with 3 stitches. S61.8 Wound of the hand.
10. 24-year-old woman, waitress. Symptoms: Ankle twisted while jogging, now painful and cannot endure standing on it. No fractures. S93.4 Sprain of the ankle.
11. 44-year-old woman, cook. Symptoms: Spilled frying fat on a thigh. You grade the burn as superficial and it covers about 5% of the surface of the body. T24.1 First grade burn of lower extremity.
12. 32-year-old man, asphalt worker. Symptoms: Itching rash in elbow folds. Wears a collar while working. L20.0 Atopic eczema.
13. 27-year-old woman, cashier. Symptoms: Uterine contractions, 20. week of pregnancy. No alarming in status. O47.0 Contractions not related to labour.
14. 55-year-old woman, secretary. Symptoms: Formerly diagnosed chronic obstructive pulmonary disease. Now has a flu and difficulty of breathing. You fix the medication. J44.0 Worsening of COPD.
15. 26-year-old woman, student, works in commercial sector. Symptoms: No former diagnosis of depression, now the criteria of moderate depression are fulfilled. You decide to treat her as an outpatient. F32.1 Moderate depression.
16. 45-year-old man, office manager. Symptoms: No former diagnosis of depression, now the criteria of moderate depression are fulfilled. You decide to treat him as an outpatient. F32.1 Moderate depression.
17. 43-year-old woman, nurse. Symptoms: Long history of short-term jobs and now that the recession is beginnig, she is worried about the continuity of her career. F43, Z73 Adaptation disorder.
18. 54-year-old man, forklift driver. Symptoms: The annual holiday is over and he’s in the binge. Patient comes to appointment at Monday when it should’ve been the first working day. Alcometer shows 0.6 permilles. R11 Nausea.
19. 22-year-old woman, hairdresser. Symptoms: Typical symptoms of urethritis, no fever. Urine sample shows signs of infection. N39.0 Urethritis.
